# Exercise training restores longevity‐associated tryptophan metabolite 3‐hydroxyanthranilic acid levels in middle‐aged adults

**DOI:** 10.1111/apha.70041

**Published:** 2025-04-03

**Authors:** Niklas Joisten, Marcel Reuter, Friederike Rosenberger, Andreas Venhorst, Marie Kupjetz, David Walzik, Alexander Schenk, Adrian McCann, Per Magne Ueland, Tim Meyer, Philipp Zimmer

**Affiliations:** ^1^ Division of Exercise and Movement Science, Institute for Sport Science University of Göttingen Göttingen Germany; ^2^ Division of Performance and Health (Sports Medicine), Institute for Sport and Sport Science TU Dortmund University Dortmund Germany; ^3^ Institute of Sports and Preventive Medicine University of Saarland Saarbrücken Germany; ^4^ German University of Applied Sciences for Prevention and Health Management Saarbrücken Germany; ^5^ Bevital AS Bergen Norway

**Keywords:** aging, exercise, high‐intensity interval training, kynurenine pathway, tryptophan

## Abstract

**Aim:**

Recent pre‐clinical evidence suggests that the tryptophan metabolite 3‐hydroxyanthranilic acid (3‐HAA) and the related enzyme activity along the kynurenine metabolic pathway (KP) are associated with lifespan extension. We aimed to translate these findings into humans and expose exercise training as a potential non‐pharmacological intervention to modulate this metabolic hub.

**Methods:**

To explore whether recent pre‐clinical findings might also be of relevance for humans, we analyzed the evolutionary conservation of KYNU and HAAO, the two core KP enzymes associated with 3‐HAA. In a cross‐sectional analysis of young‐to‐middle‐aged adults (*N* = 84), we examined potential associations of serum 3‐HAA and its precursor anthranilic acid with age. We then investigated whether 26 weeks of endurance exercise (increasing intensity (INC) during the intervention period (*n* = 17) vs. conventional moderate continuous training (CON) matched for energy expenditure (*n* = 17)) impacted 3‐HAA levels, related metabolic ratios, and other KP metabolites.

**Results:**

We demonstrate that the core KP enzymes associated with 3‐HAA are evolutionarily conserved in humans. Serum 3‐HAA and its precursor anthranilic acid were consistently associated with age in young‐to‐middle‐aged adults. Both exercise modes tested induced an increase in 3‐HAA levels of 134% (*p* < 0.001) and 85% (*p* < 0.001) compared with baseline, respectively, without a significant time*group interaction effect.

**Conclusion:**

We translate the association between systemic 3‐HAA levels and age from animal models into humans and highlight longer‐term exercise training as an efficient strategy to boost systemic 3‐HAA levels in middle‐aged adults. Our findings open promising research avenues concerning the mediating role of 3‐HAA in training adaptations, health, and longevity.

## 1 INTRODUCTION

An imbalanced tryptophan degradation along the kynurenine pathway (KP) represents a hallmark of aging and several age‐related diseases.^1^ While the initial and key regulating enzyme of the KP, indoleamine 2,3‐dioxygenase‐1 (IDO‐1), is chronically upregulated in various cell types and tissues during the aging process,^2^, ^3^ investigations across different animal models have exposed further KP enzymes as potential targets for healthy aging or lifespan extension, such as tryptophan 2,3‐dioxygenase (TDO2),^4^ kynureninase (KYNU),^5^ or aminocarboxymuconate semialdehyde decarboxylase (ACMSD).^6^ Altered KP enzyme activity results in modified KP metabolite abundance, which in turn can affect various physiological processes related to aging, including energy homeostasis, oxidative stress response, and inflammation.^7^, ^8^

Recently, the KP downstream metabolite 3‐hydroxyanthranilic acid (3‐HAA) has been shown to slow down aging and promote longevity in animal models. Dang et al.^9^ demonstrated that knockdown of the 3‐HAA 3,4‐dioxygenase gene 1 (*haao‐1*), which encodes for the enzyme that further catabolizes 3‐HAA, leads to a lifespan extension of ~30%, and contributes to healthy aging in *Caenorhabditis elegans* (*C. elegans*). The authors then investigated this observation in mice and reported that both supplementing the diet with 3‐HAA in male mice as well as knocking down *haao‐1* in female mice significantly extended lifespan compared with wild‐type mice.^9^ These results point to the KP enzyme HAAO and the KP metabolite 3‐HAA as potential therapeutic targets for age‐related health decline.

Many epidemiological studies show that physical fitness is related to longevity.^10^ In this context, endurance exercise training is a widely accepted strategy to effectively promote healthy aging.^11^ The only large‐scale randomized controlled trial (*n* = 1567), the *Generation 100 study*, investigated the effect of high‐intensity interval training (HIIT) versus moderate‐intensity continuous training (MICT) versus a control group on all‐cause mortality over 5 years as the primary endpoint. The results of this trial point to the superiority of HIIT compared with conventional MICT in lowering aging‐associated all‐cause mortality, but there was only a non‐significant trend reported for effects between HIIT and MICT.^12^ Moreover, epidemiological evidence shows that former elite athletes live substantially longer than the general population,^13^, ^14^ suggesting that training at greater volumes and/or higher intensities may contribute to longevity. Endurance training improves fat oxidation^15^ and increases cardiovascular fitness (i.e., V̇O_2max_)^16^ which leads to higher performance capacity and lower risk of lifestyle‐related diseases (e.g., type 2 diabetes). However, more exploratory translational trials are needed to improve our understanding of the mechanisms linking exercise training adaptations to the aging process, health, and longevity, and finally optimize population‐specific exercise recommendations.

There is a limited but growing body of evidence on the KP in the context of exercise, with studies demonstrating that endurance exercise training is a potent modulator of the KP in animal models and in humans.^17^ Agudelo et al.^18^ showed that an exercised skeletal muscle mediates the conversion of kynurenine to kynurenic acid, thereby protecting mice from stress‐induced depression. We and others have replicated this exercise‐induced increase in kynurenic acid levels in mice in different human populations.^17^, ^19^ However, until now, the analysis of KP metabolite levels in the context of exercise is limited to the assessment of a few key metabolites, and investigations on 3‐HAA are lacking.^17^

Here, we aimed to translate the findings from Dang et al.^9^ into humans and investigated whether (i) the KP enzymes KYNU and HAAO are evolutionarily conserved using sequence alignment, (ii) serum levels of 3‐HAA are associated with age, and (iii) exercise training can boost 3‐HAA levels in middle‐aged adults. To explore exercise intensity as a potentially relevant factor, we tested continuously increasing exercise intensity (INC) throughout a 26‐week exercise intervention period versus maintaining moderate‐intensity continuous training (CON, corresponding to MICT).

## 2 RESULTS AND DISCUSSION

### 2.1 Core kynurenine pathway enzymes associated with 3‐HAA are evolutionarily conserved

**FIGURE 1:**
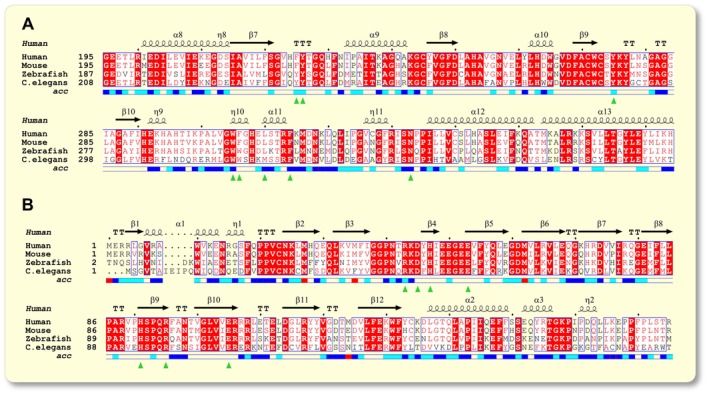
Evolutionary conservation of core kynurenine pathway enzymes associated with 3‐hydroxyanthranilic acid. A: Multiple sequence alignment of amino acid residues 195–374 of kynureninase (KYNU). B: Multiple sequence alignment of amino acid residues 1–175 of 3‐hydroxyanthranilic acid 3,4‐dioxygenase (HAAO). Full amino acid sequences are depicted in Supplement 2 and 3. Similarity between the sequences is color‐coded: Red box with white character = strict identity, red character = similarity in a group, blue frame = similarity across groups. Secondary structures are displayed above the aligned sequences and relative accessibility is color‐coded beneath: Blue = accessible, cyan = intermediate, white = buried, red = accessibility not predicted. Green triangles indicate residues involved in substrate cleavage as reported previously.^20^, ^21^, ^22^

To explore whether the pre‐clinical findings by Dang et al.^9^ might also be of relevance for humans, we analyzed the evolutionary conservation of KYNU and HAAO, the two core KP enzymes associated with 3‐HAA. KYNU catalyzes the cleavage of 3‐hydroxykynurenine (3‐HK) into 3‐HAA, and HAAO catalyzes the oxidative ring opening of 3‐HAA to 2‐amino‐3‐carboxymuconate semialdehyde, which spontaneously cyclizes to quinolinic acid (QA). Multiple sequence alignment of both KYNU and HAAO revealed high evolutionary conservation between *C. elegans*, zebrafish, mouse, and human (Figure 1). For KYNU, multiple sequence alignment revealed excellent conservation, as indicated by a total consistency value of 99 (with 100 meaning full agreement between the considered alignments and the associated primary library).^23^ Evolutionary conservation was also apparent for amino acid residues involved in the enzymatic cleavage of 3‐HK, with most residues showing strict identity across the investigated taxa (Figure 1A). These included active site residues like Tyr‐226, Tyr‐275, Trp‐305, and Phe‐314^20^, ^24^ as well as residues His‐102 and Asn‐333, which were previously described to determine the substrate specificity of KYNU for 3‐HK over kynurenine^21^ (see green triangles in Figure 1A and Supplement 2). Similarly, multiple sequence alignment revealed excellent conservation for HAAO, with a total consistency value of 99. Since docking of 3‐HAA at the active site of HAAO is dependent on iron,^22^ both residues interacting with the iron ion and residues interacting with 3‐HAA directly were assessed for evolutionary conservation. Interestingly, all residues demonstrated strict identity between the investigated taxa (see green triangles in Figure 1B). This suggests that KYNU and HAAO are evolutionary highly conserved enzymes. More importantly, the evolutionary conservation also applies to the active sites involved in substrate binding and cleavage. This places the findings obtained in *C. elegans* by Dang et al.^9^ in an interesting light and warrants evaluation of potential similar effects in humans. To contextualize the evolutionary conservation of KYNU and HAAO, it is worth noting that, to the best of our knowledge, data on a potential change in gene expression during aging are lacking.

### 2.2 Systemic 3‐HAA levels associate with age in young‐to‐middle‐aged adults

**FIGURE 2:**
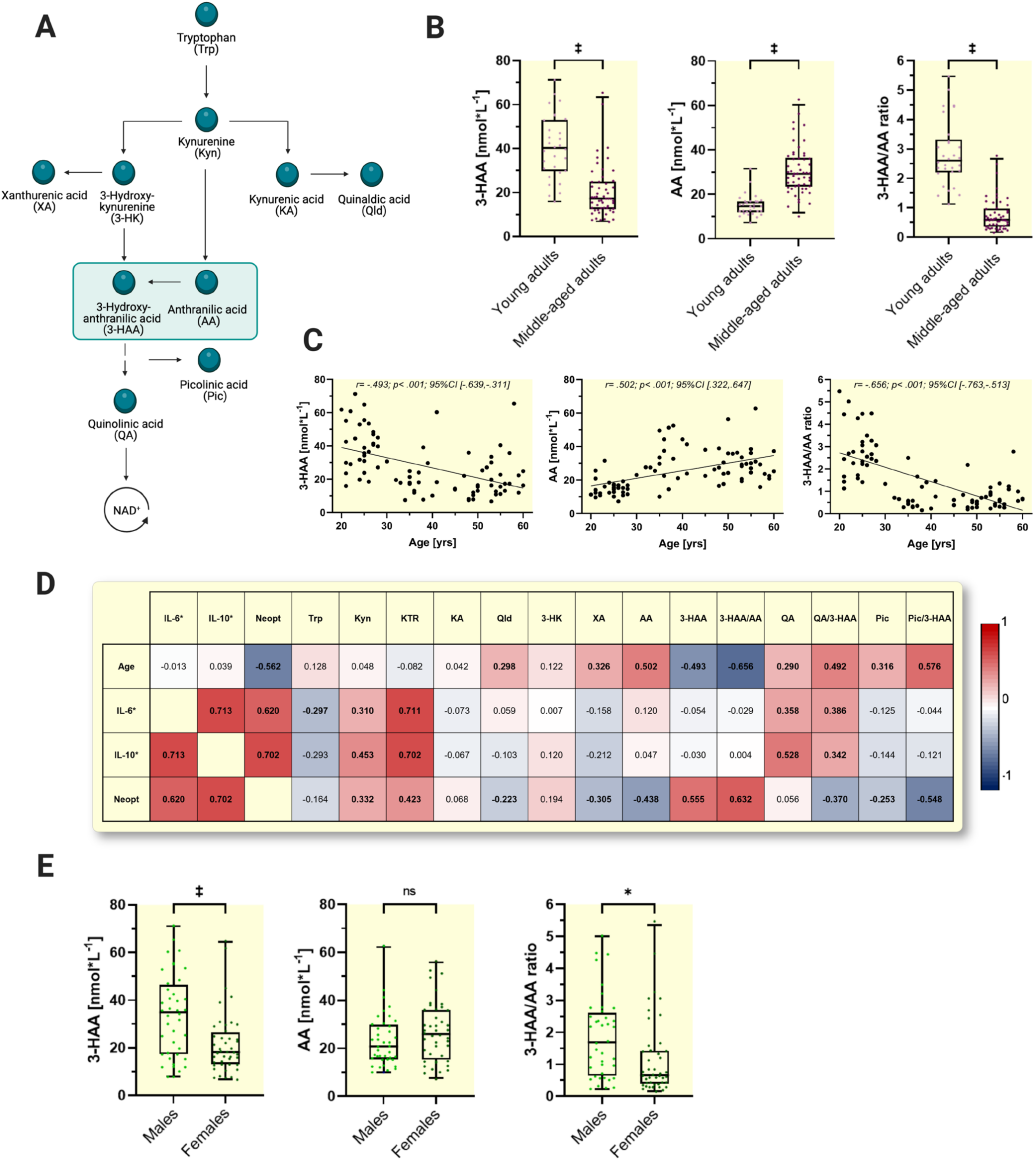
Tryptophan metabolite 3‐hydroxyanthranilic acid (3‐HAA) associates with age in young‐to‐middle‐aged adults. A: Schematic overview of the kynurenine pathway (KP) of tryptophan metabolism. B: Age‐related differences in the level of 3‐HAA, anthranilic acid (AA), and 3‐HAA/AA ratio between young (20–29 years of age, *n* = 30) and middle‐aged (30–60 years of age, *n* = 54) adults, illustrated by boxplots with 95% confidence intervals as whiskers. C: Scatter plots illustrating correlations (*r*: Pearson's coefficient; *CI*: 95% confidence interval) between age and level of 3‐HAA, AA, and 3‐HAA/AA ratio (pooled cohorts, *N* = 84). D: Heatmap showing correlations (Pearson's coefficient) between all KP metabolites, age (*N* = 84), and conventional serum inflammation markers (IL‐6: *N* = 47; IL‐10: *N* = 43). Significant correlations (*p* < 0.05) were indicated in bold. E: Sex‐related differences in levels of 3‐HAA, AA, and 3‐HAA/AA ratio (males: *N* = 40; females: *N* = 44) illustrated as boxplots with 97.5 and 2.5 percentile as whiskers. *p < 0.05; **p < 0.01; ***p < 0.001, based on independent *t*‐tests. CON = control group; INC = intervention group; IL‐6 = interleukin‐6; IL‐10 = interleukin‐10; Neopt = neopterin; Trp = tryptophan; Kyn = kynurenine; KTR = kynurenine‐to‐tryptophan ratio; KA = kynurenic acid; Qld = quinaldic acid; 3‐HK = 3‐hydroxykynurenine; XA = xanthurenic acid; AA = anthranilic acid; 3‐HAA = 3‐hydroxyanthranilic acid; QA = quinolinic acid; Pic = picolinic acid; 3‐HAA/AA ratio = 3‐hydroxyanthranilic acid/anthranilic acid ratio; QA/3‐HAA ratio = quinolinic acid/3‐hydroxyanthranilic acid ratio; Pic/3‐HAA ratio = picolinic acid/3‐hydroxyanthranilic acid ratio. yrs: Years T_0_‐T_1_: All participants completed 50 min continuous walking/cycling at 55% heart rate reserve (HR_R_)). Randomization to CON/INC was performed after 10 weeks (T_1_). CON participants continued 50 min continuous walking/cycling at 55%HR_R_ for 16 weeks (T_1_‐T_3_). INC participants completed 50 min continuous walking/cycling at 70% HR_R_ for 8 weeks (T_1_‐T_2_) and high‐intensity interval training (4 × 4 min at 95% HR_R_) for 8 weeks (T_2_ to T_3_). KTR is given in μmol*L^−1^ by mmol*L^−1^. 3‐HAA/AA ratio, QA/3‐HAA ratio, and Pic/3‐HAA ratio are given in nmol*L^−^1 by nmol*L^−1^.

To evaluate a potential relationship between systemic 3‐HAA concentrations and age in humans, we determined serum concentrations of 3‐HAA and the complete KP metabolite profile (Figure 2A) in a cohort of *N* = 84 adults (44 females) with an age range of 20–60 years (see Table 1 for participant characteristics), using a targeted metabolomics approach. We first compared systemic levels of 3‐HAA, its direct precursor anthranilic acid (AA), and the 3‐HAA/AA ratio between young (20–29 years of age) and middle‐aged (30–60 years of age) adults. Levels of 3‐HAA were twice as high in the young adults compared with the middle‐aged adults, while levels of AA were substantially lower in the young adults (Figure 2B). Accordingly, the 3‐HAA/AA ratio emerged as the marker that best discriminated between young and middle‐aged adults. The association between the AA‐3‐HAA axis (refers to AA, 3‐HAA, and the ratio 3‐HAA/AA) and age is also persistent when using both metabolite levels and their ratio as continuous variables across all *N* = 84 participants, as reflected in consistent significant correlations of 3‐HAA (*r* = −0.493; *p* < 0.001), AA (*r* = 0.502; *p* < 0.001), and 3‐HAA/AA (*r* = −0.656; *p* < 0.001) with age, respectively (Figure 2C). Conventional markers of inflammation, that is, IL‐10 and IL‐6, did not correlate with age or with components of the AA‐3‐HAA axis (Figure 2D), suggesting an inflammation‐independent mechanism may underlie the age‐associated decline in 3‐HAA concentrations, although further research including more markers of the complex picture of inflammation is needed to support this hypothesis. However, in line with the literature, both markers, IL‐10 and IL‐6, as well as neopterin correlated with the kynurenine‐to‐tryptophan ratio (KTR) as an indicator of KP activation and with quinolinic acid levels, presumably due to increased activity of the inflammation‐responsive KP enzymes, IDO‐1 and kynurenine 3‐monooxygenase (KMO), respectively.^25^, ^26^ The observed significant associations of the macrophage‐derived neopterin and the KP metabolites reflect the well‐established interconnection between neopterin and KP regulation in the present cohort of investigation.

**TABLE 1:** Participant characteristics of the cross*‐*sectional investigation.

	Overall sample	Young adults (20–29 yrs)	Middle‐aged adults (30–60 yrs)
	*N* = 84	*n* = 30	*n* = 54
Sex			
*Male*	40 (47.6%)	18 (60.0%)	22 (40.7%)
*Female*	44 (52.4%)	12 (40.0%)	32 (59.3%)
Age [years]	38.94 (13.08)	24.40 (2.42)	47.02 (8.86)
BMI [kg/m^2^]	24.44 (3.13)	23.26 (2.58)	25.09 (3.24)
Body fat [%]	21.43 (5.35)	19.65 (7.32)	22.42 (3.58)
Relative V̇O_2peak_ [mL·min^−1^·kg^−1^]	38.67 (7.97)	46.29 (7.41)	34.42 (4.26)

*Note*: Sex is given as total number and percentage (%). All other data are given as mean (standard deviation).

Abbreviations: BMI, body mass index; V̇O_2peak_, peak oxygen consumption during cardiopulmonary exercise testing by kilogram body weight.

Mechanistically, a physiological consequence of the age‐dependent decline in systemic 3‐HAA levels could be a decreased competence in oxidative stress response. Oxidative stress is closely linked to aging and several age‐related diseases. In this context, age‐associated functional impairments are attributed to an imbalance between reactive oxygen and nitrogen species production and antioxidant defense capacity.^27^ Given that 3‐HAA has been described as a context‐dependent redox regulator,^9^, ^28^ chronically decreased systemic 3‐HAA concentrations might contribute to aging processes, and interventions to counteract this decline are worth investigating.

Additionally, we evaluated potential sex differences in the systemic concentrations of the AA‐3‐HAA axis and observed significantly higher levels of 3‐HAA and the 3‐HAA/AA ratio in male compared with female participants (Figure 2E). Of note, the significant correlation between 3‐HAA level and age persisted when dichotomizing the participants by sex (Supplement 4). In their recent article, Dang et al.^9^ report that the knockout of *haao‐1*, the gene mediating the metabolic degradation of 3‐HAA, significantly extended the lifespan of female mice, whereas no significance for lifespan extension was observed in male mice. Our findings confirm a sex‐dependent metabolic regulation of systemic 3‐HAA levels in humans. The observed differences in metabolite concentrations can be explained by several potential mechanisms, such as increased/decreased enzyme activity (e.g., via post‐translational modifications or epigenetic regulation) or hormonal differences that might influence the kynurenine pathway regulation between males and females. However, based on the current investigations, the source organ of the observed KP metabolite differences between sexes remains unknown and it is thus crucial for future research to elucidate the underlying mechanisms on a tissue level.

### 2.3 Endurance exercise training over 26 weeks restores age‐related decline in systemic 3‐HAA levels

**FIGURE 3:**
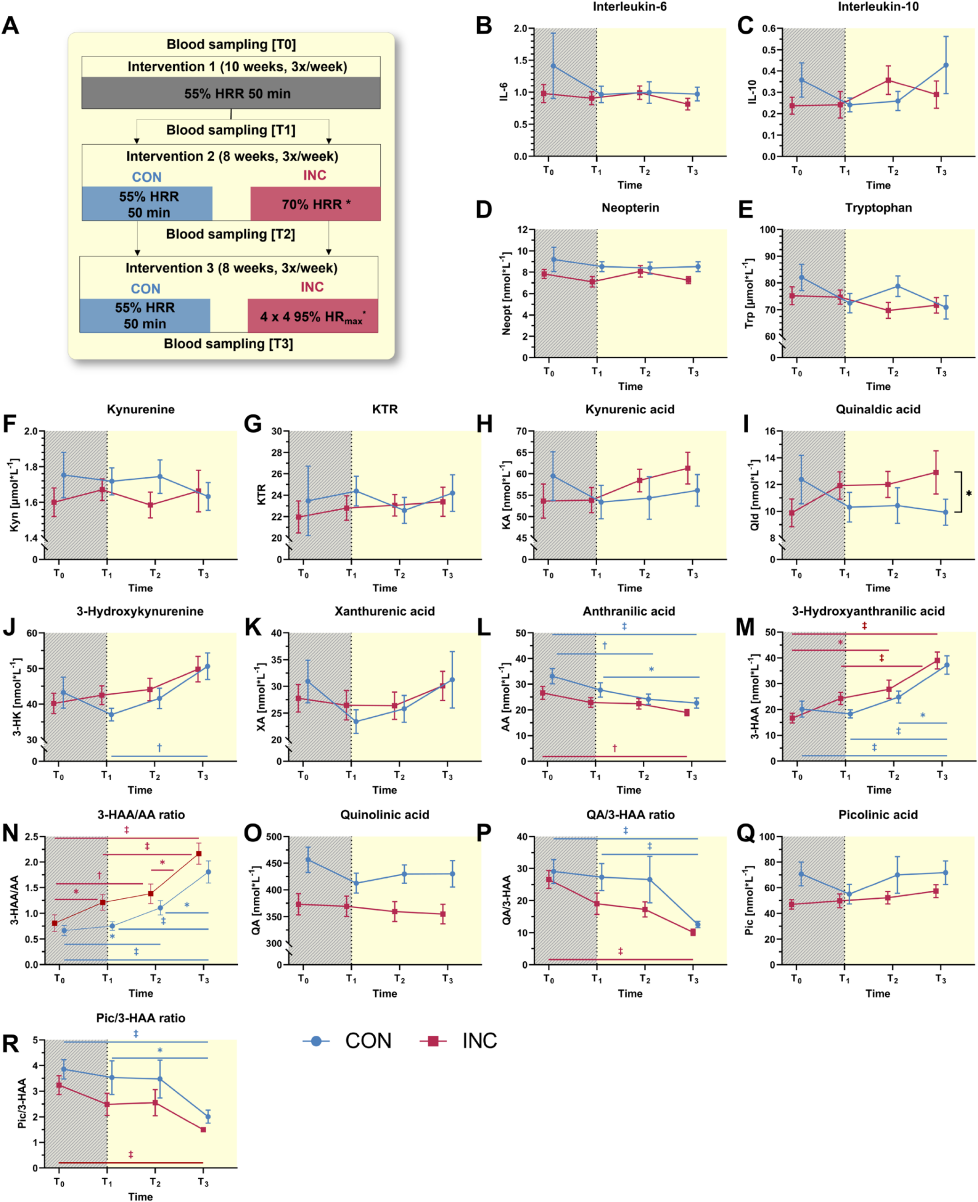
Markers of inflammation and systemic tryptophan metabolite levels along the kynurenine pathway in response to 26 weeks of either increased intensity exercise (INC) or moderate‐intensity exercise (CON). A: Study design of the randomized controlled trial investigating serum KP modulating effects of a 26‐week endurance exercise training (conventional moderate‐intensity continuous training (CON, *n* = 17) versus increasing exercise intensity training (INC, *n* = 17)) at baseline (T_0_), 10 weeks (T_1_), 18 weeks (T_2_), and 26 weeks (T_3_). The first 10 weeks of the intervention period (indicated as gray zone in the figures) were implemented as exercise familiarization phase during which all participants conducted the conventional moderate‐intensity exercise training. *Energy expenditure for INC was matched to CON by adjusting the training time per session for intervention 2 (mean duration, 42 min per session) and intervention 3 (mean duration, 35 min per session). B‐R: Results for the separate markers and metabolites. Levels of 3‐HAA increase over 26 weeks of INC by 134%, while CON induces a descriptively smaller increase (85%). Repeated measures analyses of variance (ANOVA) and Bonferroni‐corrected post hoc comparisons (in case of significant ANOVA main effects) were conducted for all analytes. Sample sizes were *n* = 17 for INC versus *n* = 17 for CON for all analytes except for IL‐10 (INC: *N* = 12; CON: *N* = 15) *significant between group post hoc comparison; #significant within group post hoc comparison. *p < 0.05; # *p* < 0.05; ##*p* < 0.01; ###*p* < 0.001. Values are reported as mean ± standard error of the mean. CON = control group; INC = intervention group; KTR = kynurenine‐to‐tryptophan ratio; 3‐HAA/AA ratio = 3‐hydroxyanthranilic acid/anthranilic acid ratio; QA/3‐HAA ratio = quinolinic acid/3‐hydroxyanthranilic acid ratio; Pic/3‐HAA ratio = picolinic acid/3‐hydroxyanthranilic acid ratio. T_0_‐T_1_: All participants completed 50 min continuous walking/cycling at 55% heart rate reserve (HR_R_)). Randomization to CON/INC was performed after 10 weeks (T_1_). CON participants continued 50 min continuous walking/cycling at 55%HR_R_ for 16 weeks (T_1_‐T_3_). INC participants completed 50 min continuous walking/cycling at 70% HR_R_ for 8 weeks (T_1_‐T_2_) and high‐intensity interval training (4 × 4 min at 95% HR_R_) for 8 weeks (T_2_ to T_3_). KTR is given in μmol*L^−1^ by mmol*L^−1^. 3‐HAA/AA ratio, QA/3‐HAA ratio, and Pic/3‐HAA ratio are given in nmol*L^−^1 by nmol*L^−1^.

Replicating the association between age and 3‐HAA levels in young‐to‐middle‐aged humans, we next asked whether long‐term endurance exercise training, as an evidence‐based strategy with multi‐dimensional health effects across the lifespan and KP modulating properties, could restore the age‐related decline in systemic 3‐HAA levels. We analyzed serum KP metabolite levels of *n* = 34 middle‐aged adults who participated in a randomized endurance exercise trial over 26 weeks at four different measurement time points (baseline: T_0_; 10 weeks: T_1_; 18 weeks: T_2_; 26 weeks: T_3_). The study was designed to compare the effects of two different exercise intensity groups (INC vs. CON) that were matched for caloric demand over the entire intervention period (Figure 3A).

Changes by exercise training were neither observed for inflammatory markers nor for KP upstream metabolites, but both training modes, INC and CON, substantially increased systemic 3‐HAA levels and concomitantly decreased systemic AA levels (Figure 3B–M). The increase in 3‐HAA levels from baseline (T0) to post‐intervention (T3) was 85% for CON and 134% for INC, without reaching a significant time*group interaction effect (Supplement 5). Of note, the 3‐HAA levels increased incrementally throughout all four measurements, with a concurrent decrease in AA levels. These findings indicate that longer‐term endurance exercise training is an efficient intervention to restore an age‐related decline in systemic 3‐HAA levels. In fact, systemic levels of 3‐HAA in both groups, INC and CON, are comparable to those of young adults (Figure 2B) after participating in the 26‐week endurance exercise program (Figure 3M). The incremental increase in 3‐HAA levels throughout the intervention period emphasizes the importance of continued long‐term endurance exercise training to achieve substantial physiological adaptations. Despite some differences in the endurance exercise training effect between INC and CON, there was no statistical significance between groups over time, suggesting an intensity‐independent effect or at least a small effect that was undetectable under the given conditions of sample size and variation. Since both interventions were matched for calories spent during the exercise intervention, the total energy expenditure during exercise appears to be more relevant for modulating 3‐HAA levels than other factors associated with training at higher intensities (e.g., distinct hormonal and metabolic response). In terms of (anti‐)inflammatory effects that have been attributed to longer‐term endurance training,^29^ we did not observe any significant change by the exercise program for IL‐6, IL‐10, or neopterin (Figure 3B–D). The boosting effect on 3‐HAA levels is therefore not mediated by lowering the inflammatory state, which represents a well‐described hallmark of aging.^30^

Speculating about other factors than inflammation potentially underlying the observed exercise‐induced increase in 3‐HAA, gut microbiota‐mediated changes in tryptophan metabolism might be one key mechanism. Exercise training has been described to alter gut microbiota in mice^31^ and humans,^32^, ^33^ and tryptophan metabolism is regulated by gut microbiota.^34^ Indeed, two studies have reported that acute (single) bouts of endurance exercise affect tryptophan biosynthesis and metabolism in fecal samples,^35^, ^36^ but no specific conclusions on the generation of 3‐HAA or enzyme activity along the KP can be drawn. Future translational studies on exercise‐induced changes in tryptophan metabolism driven by gut microbiota are warranted to improve our mechanistic understanding of alterations in KP metabolite serum levels.

### 2.4 Physiological accumulation of systemic 3‐HAA levels

In the study by Dang et al.^9^ the inhibition of *haao‐1* in female and 3‐HAA supplementation in male mice extended the lifespans compared with the wild types, respectively. Mechanistically, the authors argue that the accumulation of physiological 3‐HAA levels prolonged life by mediating anti‐inflammatory effects and protecting against oxidative stress.^9^ In fact, the lower and thus more physiological dose of 3‐HAA supplementation investigated in the mouse model was more effective in lifespan extension compared with the higher dose.^9^ To test whether the exercise training‐induced increase in 3‐HAA is driven by an overall upregulation of the KP metabolic downstream towards its end products, we evaluated the ratios between 3‐HAA and its downstream metabolites QA and picolinic acid (Pic) (Figure 3P,R). Both ratios, QA/3‐HAA and Pic/3‐HAA, considerably decreased after the 26‐week exercise training program (more than 100% relative to baseline values in INC and CON, Supplement 5). These findings suggest that an increased upstream metabolism yielding greater 3‐HAA production coupled with decreased activity of the downstream enzymes HAAO or ACSD‐1 occurs in response to endurance exercise training in humans. The physiological accumulation of 3‐HAA, induced by 26 weeks of endurance exercise, is similar to accumulated systemic 3‐HAA levels reported in the mouse model by Dang et al.^9^ after inhibition of *haao* or 3‐HAA supplementation, which extended lifespan in mice. Taken together, both the effect of 3‐HAA supplementation on lifespan in mice as well as the association of age with 3‐HAA and the boosting effect of exercise training on 3‐HAA levels in middle‐aged adults build a solid basis for large‐scale human trials investigating 3‐HAA and its role for healthy aging.

The findings of this study should be considered in the context of its limitations. First, the time‐of‐day standardization of the blood sampling was only done within each subject for the longitudinal exercise trial and not between the subjects, thus potentially leading to variations in the outcome measurements. Second, the small sample sizes represent a limitation and the results of this study need to be reproduced by powered trials. Third, we did not control for dietary intake of tryptophan, which potentially could influence the results.

## 3 CONCLUSION

In conclusion, we show that (i) KYNU and HAAO are evolutionarily conserved in humans, (ii) systemic 3‐HAA concentrations are associated with age in young‐to‐middle‐aged adults, and (iii) 3‐HAA levels differ between sexes. We also demonstrate that (iv) longer‐term energy‐equivalent endurance exercise training, independent of exercise intensity, can substantially restore age‐related declines in systemic 3‐HAA levels. Coupled with the work of Dang et al.^9^ our observations lend support to basic and clinical research aimed at elucidating tissue‐specific mechanisms underlying the dysregulation of tryptophan metabolism during aging and examine the therapeutic potential of exercise for healthy aging in humans.

## 4 MATERIALS AND METHODS

### 4.1 Sequence alignment of core kynurenine pathway enzymes associated with 3‐HAA

Evolutionary conservation of kynureninase (KYNU) and HAAO—the two core KP enzymes associated with 3‐HAA—was assessed by retrieving the amino acid sequences from UniProt^37^ for the following organisms: human (*Homo sapiens*), mouse (*Mus musculus*), zebrafish (*Danio rerio*), and nematode (*C*. *elegans*). These taxa were chosen based on their evolutionary distance to evaluate whether KYNU and HAAO are conserved across large evolutionary timespans. All amino acid sequences were then uploaded to T‐Coffee,^23^ and multiple sequence alignments were computed using the Expresso server.^38^ The resulting alignment was then transferred to the ESPript server (https://espript.ibcp.fr)^39^ to visualize evolutionary conservation. Secondary structures and relative accessibility of different amino acid residues were plotted above and below the aligned sequences, respectively. To explore the evolutionary conservation of the active site of both proteins, we extracted amino acid residues involved in substrate binding and cleavage from previous publications and highlighted these residues with green triangles throughout the figures. For KYNU, an in‐depth characterization of the active site of human KYNU was taken as reference.^20^ For HAAO, a recent characterization of the active site in *Cupriavidus metallidurans* was used^22^ and the residues involved in substrate binding were aligned with a human sequence of HAAO to identify the corresponding residues in humans.

### 4.2 Study design

We first cross‐sectionally compared systemic tryptophan metabolite levels from a cohort of participants aged between 20 and 29 years to the levels from a cohort of participants aged between 30 and 60 years. The age span of the young adults was chosen in order to ensure a reliable comparator group that is free of initiated biological aging processes and age‐related declines in cardiorespiratory fitness. Detailed participant characteristics are provided in Table 1. We then pooled the participants from both cohorts (age range from 20 to 60 years) to calculate correlations between age, markers of inflammation (IL‐6, IL‐10, neopterin), and tryptophan metabolites. Finally, a cross‐sectional comparison was used to evaluate possible sex‐related differences in 3‐HAA levels, AA levels, and the 3‐HAA/AA ratio.

Next, we aimed to investigate the effects of longer‐term endurance exercise training on tryptophan metabolites along the KP and related inflammatory markers. For this purpose, we analyzed serum samples from *n* = 34 participants with an age between 30 and 60 years who participated in a two‐arm randomized controlled trial. The primary endpoint of this interventional trial was cardiorespiratory fitness as indicated by maximum oxygen consumption and previously published elsewhere.^40^ Included were 30–60 years old and untrained (last 6 months: < 1 h·wk^−1^ endurance‐type physical activity) non‐smokers. Exclusion criteria were BMI > 30 kg·m^−2^, resting blood pressure (RR_rest_) ≥ 160/100 mmHg, total cholesterol ≥300 mg·dl^−1^, maximum oxygen uptake (VO_2max_) > 50 mL·kg^−1^·min^−1^ for men; > 45 mL·kg^−1^·min^−1^ for women, iron deficiency (Ferritin ≤34 ng·mL^−1^), thyroid dysfunction (TSH ≤0.34 mU·l^−1^ ≥ 4.0 ng·mL^−1^), and medications with potential influence on target parameters (e.g., beta‐blockers) and pregnancy. Participants (see Table 2 for characteristics) in one arm performed continuous moderate‐intensity endurance training for 26 weeks (CON), while those in the other arm increased their training intensity from moderate to vigorous intensity after 10 weeks and to high‐intensity interval training after 18 weeks (INC). The CON group was designed to meet the WHO guidelines for aerobic physical activity, representing the current standard care as recommend by the WHO.^41^ Considering the accumulating evidence on the superior effects of high‐intensity training on various health‐related outcomes, we tested the CON group versus a group in which exercise intensity was increased throughout the intervention phase (INC) as novel intervention. Within‐subject energy expenditure was kept constant throughout the study by measuring oxygen uptake at training heart rates and subsequent adjustment of training durations (see Supplement 1 for energy expenditure data). Measurement time points were implemented at baseline (T_0_) and after 10 (T_1_), 18 (T_2_), and 26 (T_3_) weeks of training. The subjects were allocated to either CON or INC using a minimization technique. Stratification characteristics were age, sex, baseline V̇O_2max_, V̇O_2max_ response at T_1_ (yes/no), and the magnitude of V̇O_2max_ response at T_1_. All participants provided written informed consent. The study was approved by the Ethics Committee of the Medical Association of Saarland (registration number 219/19), and the study procedures complied with the Declaration of Helsinki.

**TABLE 2:** Participant characteristics of the longitudinal exercise trial.

	Overall sample	INC	CON
	*N* = 34	*n* = 17	*n* = 17
Sex			
*Male*	15 (44.1%)	8 (47.1%)	7 (41.2%)
*Female*	19 (55.9%)	9 (52.9%)	10 (58.8%)
Age [years]	46.41 (8.26)	45.65 (9.00)	47.18 (7.64)
BMI [kg/m^2^]	25.76 (3.36)	25.84 (3.48)	25.69 (3.34)
Body fat [%]	22.91 (3.61)	22.55 (3.66)	23.27 (3.63)
Relative V̇O_2peak_ [mL·min^−1^·kg^−1^]	34.49 (4.31)	34.19 (5.13)	34.79 (3.46)

*Note*: Sex is given as the total number and percentage (%). All other data are given as mean (SD).

Abbreviations: BMI, body mass index; CON, control group; INC, intervention group; V̇O_2peak_, peak oxygen consumption during cardiopulmonary exercise testing by kilogram body weight.

### 4.3 Testing procedures and blood sampling

A detailed description of cardiorespiratory test procedures is published elsewhere.^42^ In brief, each participant completed six tests; three tests at baseline; and one at T_1_, T_2_, and T_3_. Before exercise testing, anthropometric data and hemodynamic characteristics at rest were measured. Body fat percentage was assessed by a 10‐site skinfold method with a Harpenden caliper. Exercise tests were performed on a treadmill. The test protocol consisted of a combination of a graded exercise test (GXT) and a ramp protocol. This allowed for the measurement of submaximal parameters such as heart rate (HR) and lactate, running economy (RE), as well as maximal velocity (V_max_) and VO_2max_.^42^

Venus blood samples were collected under resting conditions (participants were strictly instructed to refrain from any exercise or physical activity inducing cardiovascular arousal for at least 24 h prior to the testings) at baseline, T_1_, T_2_, and T_3_ from a median cubital vein in the forearm after local skin disinfection in a lying position using serum tubes. After clotting for 10 minutes, samples were centrifuged at 4300*g* for 10 minutes and isolated serum was stored at −80°C until further analysis.

### 4.4 Training program

The training program was conducted 3d wk^−1^ for a duration of 26 weeks. For the initial 10 weeks, all participants trained 50 minutes per session at 55% heart rate reserve (%HR_RES_). We implemented the 10‐week familiarization phase due to several reasons. First, to investigate conventional endurance training as recommended by the WHO physical activity guidelines (CON group) versus a promising novel training method (increasing intensity, INC group), it is plausible to train all participants initially at one similar intensity in order to create a similar baseline level. Second, all participants were familiarized with the training sessions and context conditions itself. Third, the familiarization phase was used to accurately stratify the randomization (“change in VO2max” and “response/non‐response” were used as stratification factors). After the initial 10 weeks, CON continued to exercise 3d wk^−1^ for 50 minutes per session at 55%HR_RES_ until the end of the study. INC increased the intensity to 70% HR_RES_ after 10 weeks and performed a high‐intensity interval training (HIIT 4 × 4 protocol) after 18 weeks. The HIIT program consisted of a 10‐minute warm‐up at 70% HR_max_, followed by 4 intervals of 4‐minute at 95% HR_max_, interspersed with 3‐minute rest intervals at 70% HR_max_, and a cool‐down at 70% HR_max_. We decided to use heart rate reserve to control exercise intensity because this parameter is integrative as it includes both the maximum heart rate as well as the resting heart rate, while the latter represents an indicator of cardiorespiratory fitness. The duration of the training sessions was adjusted after T_1_ to maintain constant within‐subject energy expenditure.

### 4.5 Enzyme‐linked immunosorbent assays (ELISAs)

Il‐6 and IL‐10 were measured using commercial high‐sensitive ELISA kits (R&D Systems). Assays were conducted in accordance with the manufacturer's instructions.

### 4.6 Targeted metabolomics

Neopterin and the KP metabolites were measured by liquid chromatography–tandem mass spectrometry (LC–MS/MS) as described previously^43^ at the Bevital laboratory (https://bevital.no). The lower limit of detection (LOD) for the assay ranged from 0.01 to 8 nmol/L, and within‐ and between‐day coefficients of variation (CVs) ranged from 3% to 8% and 4% to 10%, respectively.

### 4.7 Statistical analyses

Correlations between serum analytes and age were calculated using Pearson's coefficient. Independent t‐tests were conducted for comparisons between young‐ and middle‐aged adults as well as between females and males. Repeated measures analyses of variance (ANOVAs) were used for statistical evaluation of within‐ and between‐group changes in all serum analytes over the intervention period. In case of significant ANOVA effects, Bonferroni‐corrected post hoc comparisons were applied. Level of significance was set at 5% for the α‐error. Two‐sided tests were used for all statistical analyses. All statistical tests were conducted using IBM SPSS statistics version 29. Line charts and box plots were created with GraphPad (version 9.1.2, GraphPad Software, San Diego, CA, USA).

## AUTHOR CONTRIBUTIONS

**Niklas Joisten:** Conceptualization; methodology; formal analysis; writing – original draft. **Marcel Reuter:** Investigation; data curation; writing – original draft. **Friederike Rosenberger:** Supervision; writing – review and editing. **Andreas Venhorst:** Supervision; writing – review and editing. **Marie Kupjetz:** Formal analysis; visualization; writing – review and editing. **David Walzik:** Formal analysis; visualization; writing – original draft. **Alexander Schenk:** Visualization; investigation; writing – review and editing. **Adrian McCann:** Investigation; resources; writing – review and editing. **Per Magne Ueland:** Resources; writing – review and editing. **Tim Meyer:** Conceptualization; methodology; validation; supervision; project administration; writing – review and editing; resources. **Philipp Zimmer:** Conceptualization; methodology; supervision; resources; writing – review and editing; project administration.

## FUNDING INFORMATION

This study was conducted without external funding.

## CONFLICT OF INTEREST STATEMENT

The authors declare that there is no conflict of interest.

## Supporting information


Data S1.

## Data Availability

All data underlying this study will be made available upon reasonable request by the corresponding author.

## References

[1] Savitz J . The kynurenine pathway: a finger in every pie. Mol Psychiatry. 2020;25:131‐147. 30980044 10.1038/s41380-019-0414-4 PMC6790159

[2] Pertovaara M , Raitala A , Lehtimäki T , et al. Indoleamine 2,3‐dioxygenase activity in nonagenarians is markedly increased and predicts mortality. Mech Ageing Dev. 2006;127:497‐499. 16513157 10.1016/j.mad.2006.01.020

[3] Frick B , Schroecksnadel K , Neurauter G , Leblhuber F , Fuchs D . Increasing production of homocysteine and neopterin and degradation of tryptophan with older age. Clin Biochem. 2004;37:684‐687. 15302611 10.1016/j.clinbiochem.2004.02.007

[4] Oxenkrug GF , Navrotskaya V , Voroboyva L , Summergrad P . Extension of life span of *Drosophila melanogaster* by the inhibitors of tryptophan‐kynurenine metabolism. Fly (Austin). 2011;5:307‐309. 22041575 10.4161/fly.5.4.18414 PMC3266072

[5] Sutphin GL , Backer G , Sheehan S , et al. *Caenorhabditis elegans* orthologs of human genes differentially expressed with age are enriched for determinants of longevity. Aging Cell. 2017;16:672‐682. 28401650 10.1111/acel.12595 PMC5506438

[6] Katsyuba E , Mottis A , Zietak M , et al. De novo NAD+ synthesis enhances mitochondrial function and improves health. Nature. 2018;563:354‐359. 30356218 10.1038/s41586-018-0645-6 PMC6448761

[7] Salminen A . Role of indoleamine 2,3‐dioxygenase 1 (IDO1) and kynurenine pathway in the regulation of the aging process. Ageing Res Rev. 2022;75:101573. 35085834 10.1016/j.arr.2022.101573

[8] Kaiser H , Parker E , Hamrick MW . Kynurenine signaling through the aryl hydrocarbon receptor: implications for aging and healthspan. Exp Gerontol. 2020;130:110797. 31786316 10.1016/j.exger.2019.110797 PMC7899131

[9] Dang H , Castro‐Portuguez R , Espejo L , et al. On the benefits of the tryptophan metabolite 3‐hydroxyanthranilic acid in *Caenorhabditis elegans* and mouse aging. Nat Commun. 2023;14(1):8338. doi:10.1038/s41467-023-43527-1 38097593 PMC10721613

[10] Clausen JSR , Marott JL , Holtermann A , Gyntelberg F , Jensen MT . Midlife cardiorespiratory fitness and the long‐term risk of mortality. J Am Coll Cardiol. 2018;72:987‐995. 30139444 10.1016/j.jacc.2018.06.045

[11] Garatachea N , Pareja‐Galeano H , Sanchis‐Gomar F , et al. Exercise attenuates the major hallmarks of aging. Rejuvenation Res. 2015;18:57‐89. 25431878 10.1089/rej.2014.1623 PMC4340807

[12] Stensvold D , Viken H , Steinshamn SL , et al. Effect of exercise training for five years on all cause mortality in older adults—the generation 100 study: randomised controlled trial. BMJ. 2020;371:m3485. doi:10.1136/bmj.m3485 33028588 PMC7539760

[13] Sarna S , Sahi T , Koskenvuo M , Kaprio J . Increased life expectancy of world class male athletes. Med Sci Sports Exerc. 1993;25:237‐244. 8450727

[14] Garatachea N , Santos‐Lozano A , Sanchis‐Gomar F , et al. Elite athletes live longer than the general population: a meta‐analysis. Mayo Clin Proc. 2014;89:1195‐1200. 25128074 10.1016/j.mayocp.2014.06.004

[15] Astorino TA , Schubert MM . Changes in fat oxidation in response to various regimes of high intensity interval training (HIIT). Eur J Appl Physiol. 2018;118:51‐63. 29124325 10.1007/s00421-017-3756-0

[16] Helgerud J , Høydal K , Wang E , et al. Aerobic high‐intensity intervals improve V˙O2max more than moderate training. Med Sci Sports Exerc. 2007;39:665‐671. 17414804 10.1249/mss.0b013e3180304570

[17] Joisten N , Kummerhoff F , Koliamitra C , et al. Exercise and the kynurenine pathway: current state of knowledge and results from a randomized cross‐over study comparing acute effects of endurance and resistance training. Exerc Immunol Rev. 2020;26:24‐42. 32139353

[18] Agudelo LZ , Femenía T , Orhan F , et al. Skeletal muscle PGC‐1α1 modulates kynurenine metabolism and mediates resilience to stress‐induced depression. Cell. 2014;159:33‐45. 25259918 10.1016/j.cell.2014.07.051

[19] Schlittler M , Goiny M , Agudelo LZ , et al. Endurance exercise increases skeletal muscle kynurenine aminotransferases and plasma kynurenic acid in humans. Am J Physiol‐Cell Physiol. 2016;310:C836‐C840. 27030575 10.1152/ajpcell.00053.2016

[20] Lima S , Khristoforov R , Momany C , Phillips RS . Crystal structure of *Homo sapiens* kynureninase. Biochemistry. 2007;46:2735‐2744. 17300176 10.1021/bi0616697 PMC2531291

[21] Phillips RS . Structure, mechanism, and substrate specificity of kynureninase. Biochim Biophys Acta. 2011;1814:1481‐1488. 21167323 10.1016/j.bbapap.2010.12.003 PMC3102132

[22] Wang Y , Liu KF , Yang Y , Davis I , Liu A . Observing 3‐hydroxyanthranilate‐3,4‐dioxygenase in action through a crystalline lens. Proc Natl Acad Sci. 2020;117:19720‐19730. 32732435 10.1073/pnas.2005327117 PMC7443976

[23] Di Tommaso P , Moretti S , Xenarios I , et al. T‐coffee: a web server for the multiple sequence alignment of protein and RNA sequences using structural information and homology extension. Nucleic Acids Res. 2011;39:W13‐W17. 21558174 10.1093/nar/gkr245 PMC3125728

[24] Phillips RS . Structure and mechanism of kynureninase. Arch Biochem Biophys. 2014;544:69‐74. 24200862 10.1016/j.abb.2013.10.020

[25] Kindler J , Lim CK , Weickert CS , et al. Dysregulation of kynurenine metabolism is related to proinflammatory cytokines, attention, and prefrontal cortex volume in schizophrenia. Mol Psychiatry. 2020;25:2860‐2872. 30940904 10.1038/s41380-019-0401-9 PMC7577855

[26] Joisten N , Ruas JL , Braidy N , Guillemin GJ , Zimmer P . The kynurenine pathway in chronic diseases: a compensatory mechanism or a driving force? Trends Mol Med. 2021;27:946‐954. 34373202 10.1016/j.molmed.2021.07.006

[27] Liguori I , Russo G , Curcio F , et al. Oxidative stress, aging, and diseases. Clin Interv Aging. 2018;13:757‐772. 29731617 10.2147/CIA.S158513 PMC5927356

[28] Christen S , Peterhans E , Stocker R . Antioxidant activities of some tryptophan metabolites: possible implication for inflammatory diseases. Proc Natl Acad Sci USA. 1990;87:2506‐2510. 2320571 10.1073/pnas.87.7.2506 PMC53718

[29] Gleeson M , Bishop NC , Stensel DJ , Lindley MR , Mastana SS , Nimmo MA . The anti‐inflammatory effects of exercise: mechanisms and implications for the prevention and treatment of disease. Nat Rev Immunol. 2011;11:607‐615. 21818123 10.1038/nri3041

[30] Ferrucci L , Fabbri E . Inflammageing: chronic inflammation in ageing, cardiovascular disease, and frailty. Nat Rev Cardiol. 2018;15:505‐522. 30065258 10.1038/s41569-018-0064-2 PMC6146930

[31] Evans CC , LePard KJ , Kwak JW , et al. Exercise prevents weight gain and alters the gut microbiota in a mouse model of high fat diet‐induced obesity. PLoS One. 2014;9:e92193. 24670791 10.1371/journal.pone.0092193 PMC3966766

[32] Allen JM , Mailing LJ , Niemiro GM , et al. Exercise alters gut microbiota composition and function in lean and obese humans. Med Sci Sports Exerc. 2018;50:747‐757. 29166320 10.1249/MSS.0000000000001495

[33] Motiani KK , Collado MC , Eskelinen JJ , et al. Exercise training modulates gut microbiota profile and improves Endotoxemia. Med Sci Sports Exerc. 2020;52:94‐104. 31425383 10.1249/MSS.0000000000002112 PMC7028471

[34] Agus A , Planchais J , Sokol H . Gut microbiota regulation of tryptophan metabolism in health and disease. Cell Host Microbe. 2018;23:716‐724. 29902437 10.1016/j.chom.2018.05.003

[35] Tabone M , Bressa C , García‐Merino JA , et al. The effect of acute moderate‐intensity exercise on the serum and fecal metabolomes and the gut microbiota of cross‐country endurance athletes. Sci Rep. 2021;11:3558. 33574413 10.1038/s41598-021-82947-1 PMC7878499

[36] Zhao X , Zhang Z , Hu B , Huang W , Yuan C , Zou L . Response of gut microbiota to metabolite changes induced by endurance exercise. Front Microbiol. 2018;9:765. 29731746 10.3389/fmicb.2018.00765 PMC5920010

[37] The UniProt Consortium . UniProt: the universal protein knowledgebase in 2023. Nucleic Acids Res. 2023;51(D1):D523‐D531. doi:10.1093/nar/gkac1052 36408920 PMC9825514

[38] Armougom F , Moretti S , Poirot O , et al. Expresso: automatic incorporation of structural information in multiple sequence alignments using 3D‐coffee. Nucleic Acids Res. 2006;34:W604‐W608. 16845081 10.1093/nar/gkl092 PMC1538866

[39] Robert X , Gouet P . Deciphering key features in protein structures with the new ENDscript server. Nucleic Acids Res. 2014;42:W320‐W324. 24753421 10.1093/nar/gku316 PMC4086106

[40] Reuter M , Rosenberger F , Barz A , et al. Does higher intensity increase the rate of responders to endurance training when total energy expenditure remains constant? A randomized controlled trial. Sports Med Open. 2023;9(1):35. doi:10.1186/s40798-023-00579-3 37209213 PMC10199994

[41] Bull FC , al‐Ansari SS , Biddle S , et al. World Health Organization 2020 guidelines on physical activity and sedentary behaviour. Br J Sports Med. 2020;54:1451‐1462. 33239350 10.1136/bjsports-2020-102955 PMC7719906

[42] Meyer T , Auracher M , Heeg K , Urhausen A , Kindermann W . Does cumulating endurance training at the weekends impair training effectiveness? Eur J Cardiovasc Prev Rehabil. 2006;13:578‐584. 16874148 10.1097/01.hjr.0000198921.34814.4d

[43] Midttun Ø , Hustad S , Ueland PM . Quantitative profiling of biomarkers related to B‐vitamin status, tryptophan metabolism and inflammation in human plasma by liquid chromatography/tandem mass spectrometry. Rapid Commun Mass Spectrom. 2009;23:1371‐1379. 19337982 10.1002/rcm.4013

