# Supplementary material for: Exercise training restores longevity‐associated tryptophan metabolite 3‐hydroxyanthranilic acid levels in middle‐aged adults
Source: Acta Physiol (Oxf). 2025 Apr 3;241(5):e70041. doi: 10.1111/apha.70041 (PMC11967295; doi:10.1111/apha.70041)
Supplement: Supplementary file 1 — Data S1. [file APHA-241-e70041-s001.pdf]

Supplements

Exercise training restores longevity-associated tryptophan metabolite 3-hydroxyanthranilic acid levels in middle-aged adults.

Short title: Exercise restores 3-hydroxyantranilic acid

Niklas Joisten[1,2*], Marcel Reuter[3,4*], Friederike Rosenberger[4], Andreas Venhorst[3], Marie Kupjetz[2], David Walzik[2], Alexander Schenk[2], Adrian McCann[5], Per Magne Ueland[5], Tim Meyer[3#], Philipp Zimmer[2#]

[1] Division of Exercise and Movement Science, Institute for Sport Science, University of Göttingen, Göttingen, Germany

[2] Division of Performance and Health (Sports Medicine), Institute for Sport and Sport Science, TU Dortmund University, Dortmund, Germany

[3] Institute of Sports and Preventive Medicine, University of Saarland, Saarbrücken, Germany

[4] German University of Applied Sciences for Prevention and Health Management, Saarbrücken, Germany

[5] Bevital AS, Bergen, Norway

[*]shared first authorship

[#]shared last authorship

Correspondence: Philipp Zimmer, philipp.zimmer@tu-dortmund.de

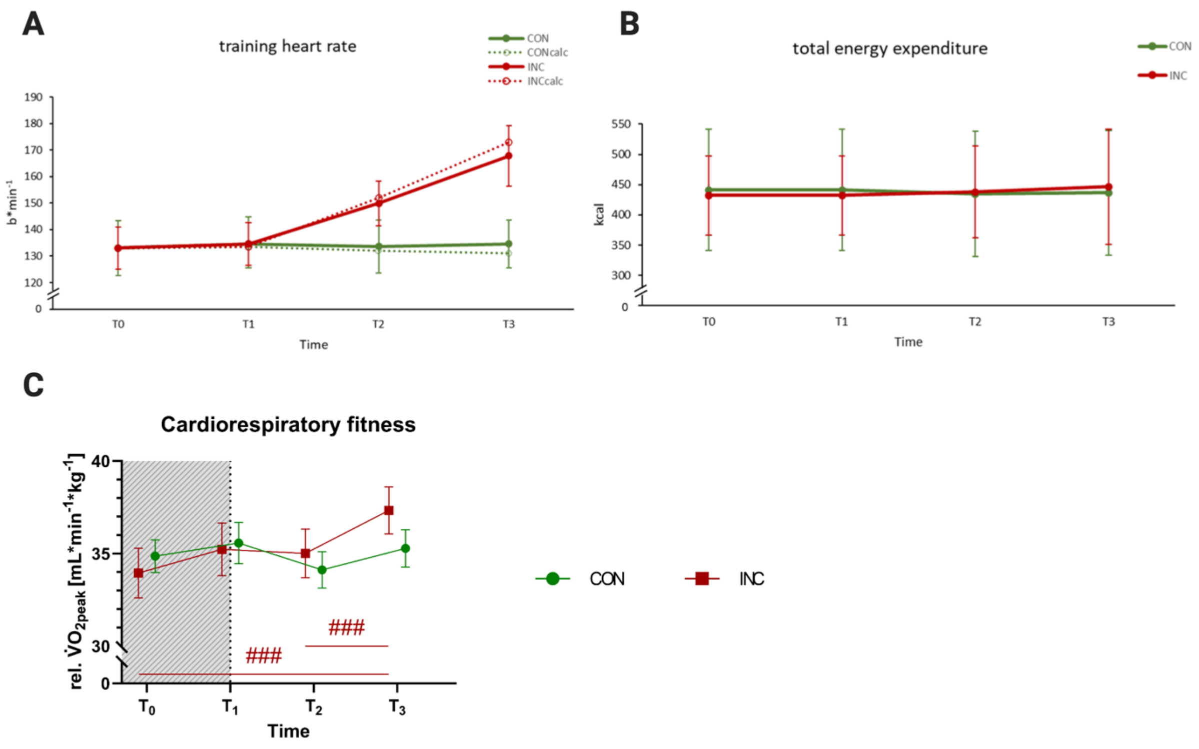

Supplement 1. Training intervention data on (A) heart rate, (B) total energy expenditure during the exercise intervention, and (C) cardiorespiratory fitness. Data from Reuter et al. 2023[1].

(A) CONcalc/INCcalc=calculated training heart rate based on the VO2peak tests. CON/INC=actual training heart rate. (B) The average energy expenditure was estimated using indirect calorimetry.

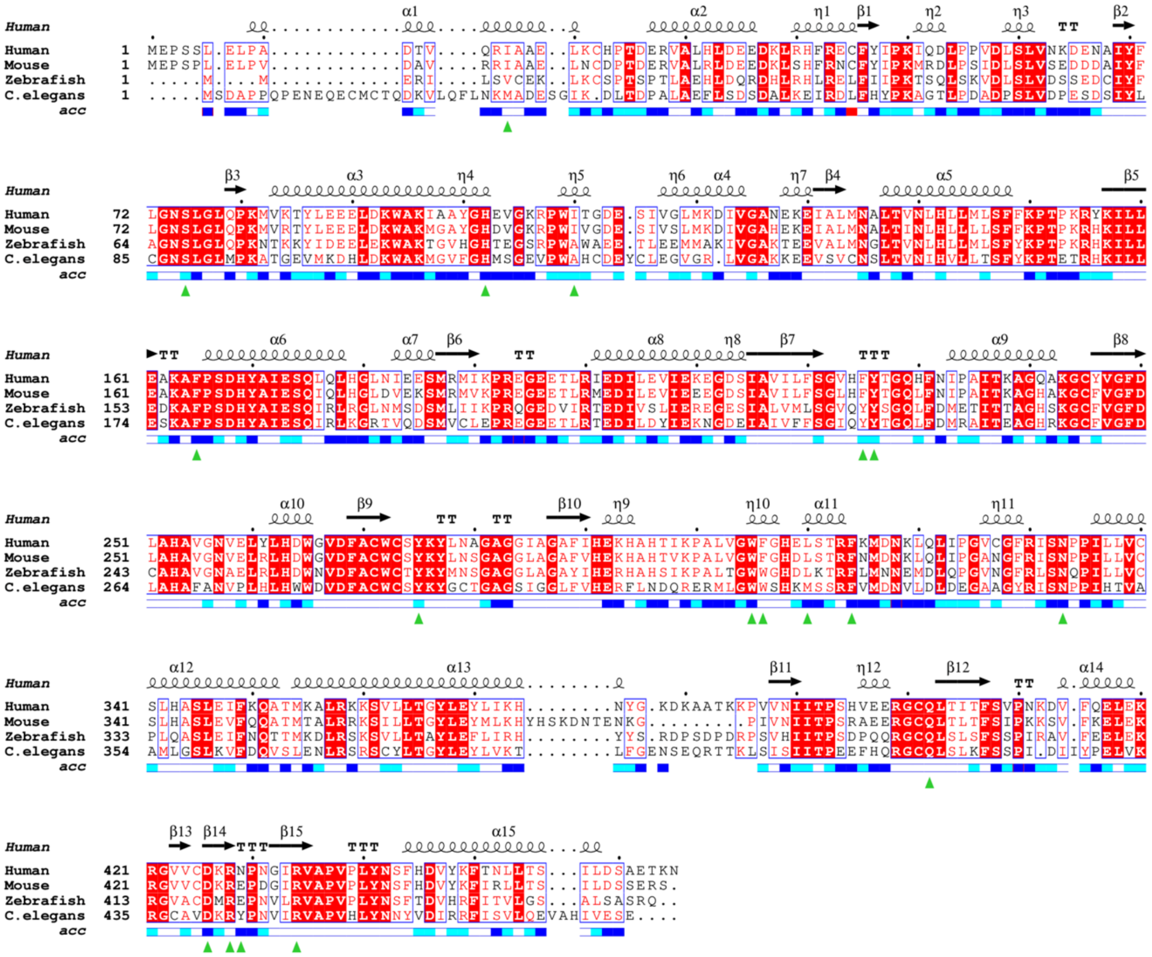

Supplement 2. Evolutionary conservation of kynureninase (KYNU). Similarity between the sequences is color-coded: red box with white character = strict identity, red character = similarity in a group, blue frame = similarity across groups. Secondary structures are displayed above the aligned sequences and relative accessibility is color-coded beneath: blue = accessible, cyan = intermediate, white = buried, red = accessibility not predicted. Green triangles indicate residues involved in substrate cleavage as reported previously[2,3].

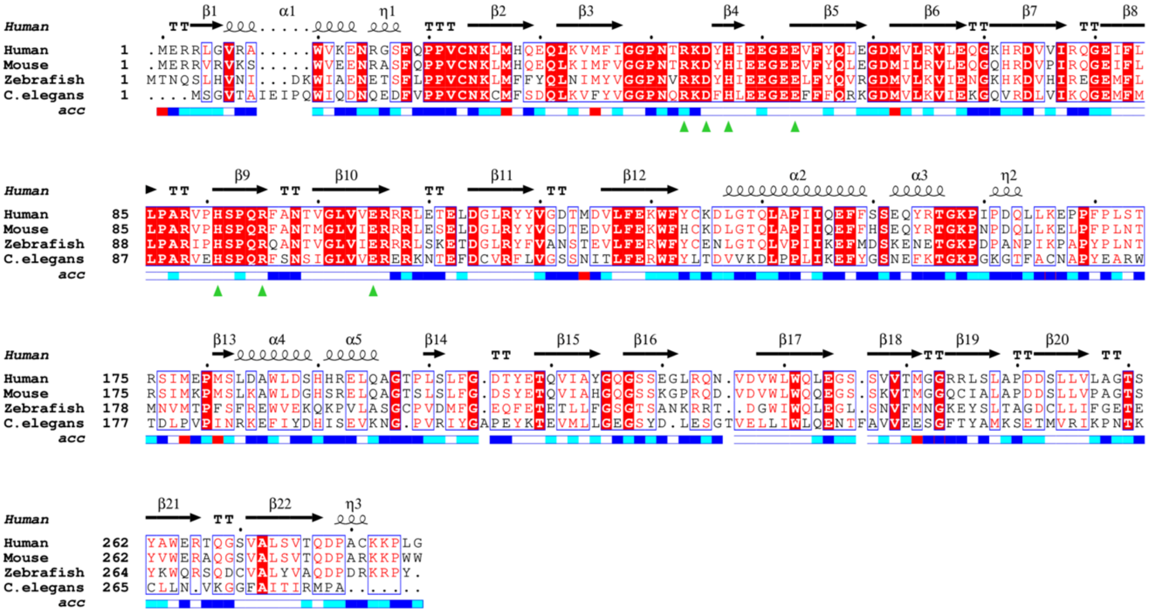

Supplement 3. Evolutionary conservation of 3-hydroxyanthranilic acid 3,4-dioxygenase (HAAO). Similarity between the sequences is color-coded: red box with white character = strict identity, red character = similarity in a group, blue frame = similarity across groups. Secondary structures are displayed above the aligned sequences and relative accessibility is color-coded beneath: blue = accessible, cyan = intermediate, white = buried, red = accessibility not predicted. Green triangles indicate residues involved in substrate cleavage as reported previously[4].

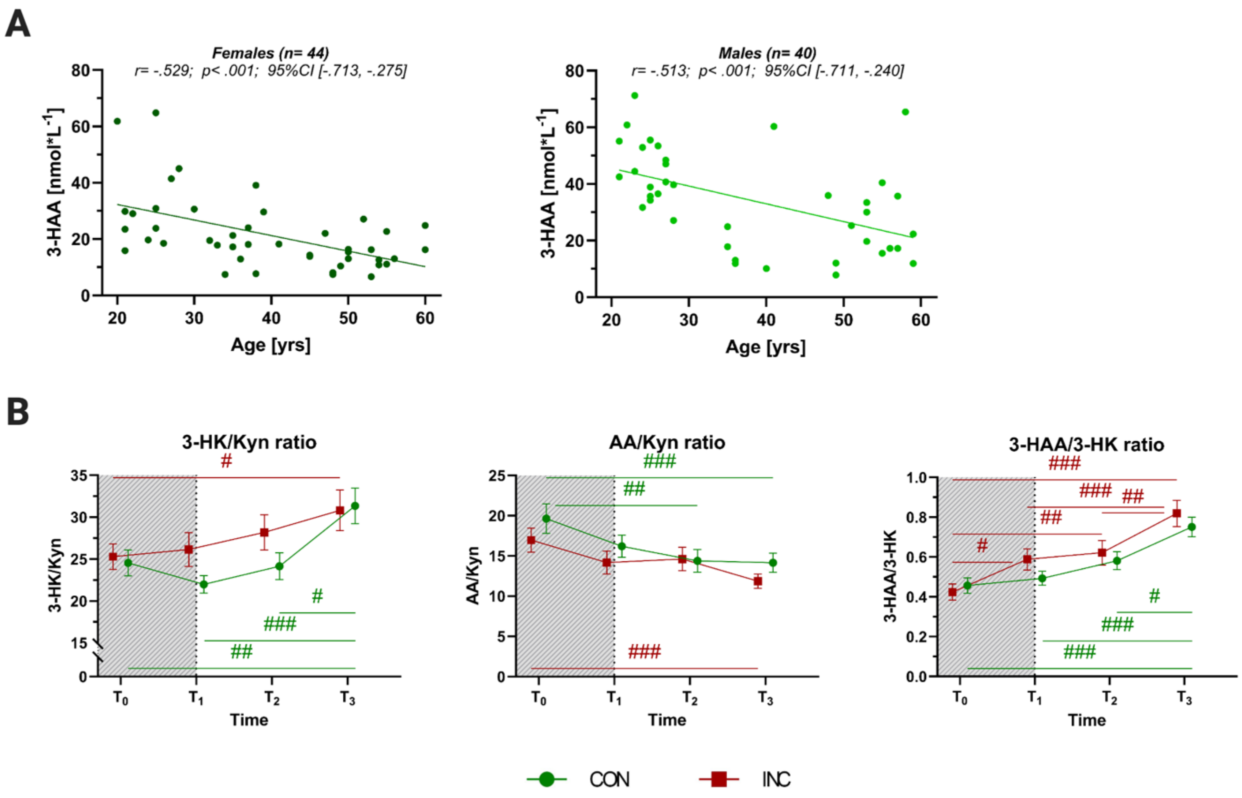

Supplement 4. (A) Systemic 3-hydroxyanthranilic acid (3-HAA) levels correlate (Pearson's coefficient) with age within female and male participants. (B) Kynurenine pathway ratios towards 3-HAA in response to 26 weeks exercise training between CON and INC.

Supplement 5. Detailed ANOVA results for all outcome measures investigated in response to 26-week endurance training (CON versus INC).

	CON				*INC*				*ANOVA*	
	T_0	*T_1 (10 wks)*	*T_2 (18 wks)*	*T_3 (26 wks)*	*T_0*	*T_1 (10 wks)*	*T_2 (18 wks)*	*T_3 (26 wks)*	*time*group*	*time*
Inflamm. Markers										
IL-6 [pg*mL^{-1}]	1.41	.97	1.00	.97	.98	.91	.99	.81	.574	.396
	(2.11)	(.53)	(.70)	(.44)	(.59)	(.41)	(.44)	(.38)		
IL-10 [pg*mL^{-1}]	.36	.24	.26	.43	.24	.24	.36	.29	.263	.356
	(.31)	(.12)	(.17)	(.52)	(.14)	(.21)	(.23)	(.22)		
Neopt [nmol*L^{-1}]	9.21	8.52	8.38	8.53	7.84	7.10	8.07	7.26	.488	.327
	(4.71)	(1.94)	(2.34)	(1.95)	(1.76)	(1.98)	(2.22)	(1.31)		
Kynurenines										
Trp [µmol*L^{-1}]	82.05	72.42	78.75	70.86	75.21	74.68	69.72	71.64	.147	.087
	(20.16)	(14.86)	(15.85)	(18.01)	(13.78)	(10.98)	(12.34)	(11.87)		
Kyn [µmol*L^{-1}]	1.75	1.72	1.74	1.63	1.60	1.67	1.59	1.66	.277	.869
	(.53)	(.31)	(.39)	(.32)	(.33)	(.25)	(.30)	(.48)		
KTR	23.48	24.38	22.58	24.19	21.96	22.79	23.06	23.39	.709	.651
	(13.41)	(5.71)	(5.02)	(7.02)	(6.20)	(4.71)	(4.12)	(5.65)		
KA [nmol*L^{-1}]	59.48	53.39	54.34	56.12	53.62	53.85	58.44	61.34	.211	.363
	(23.52)	(16.08)	(20.52)	(15.16)	(16.38)	(12.14)	(10.97)	(15.29)		
Qld [nmol*L^{-1}]	12.38	10.31	10.42	9.93	9.88	11.92	12.00	12.91	**.030***	.988
	(7.46)	(4.56)	(5.47)	(3.99)	(4.28)	(4.21)	(4.05)	(6.67)		
3-HK [nmol*L^{-1}]	43.27	37.08	41.61	50.64	40.21	42.52	44.09	49.85	.349	**<.001*****
	(17.95)	(7.20)	(11.73)	(15.55)	(11.86)	(10.76)	(12.99)	(14.92)		
XA [nmol*L^{-1}]	30.91	23.41	25.80	31.25	27.76	26.45	26.38	30.08	.614	.102
	(16.44)	(9.10)	(10.31)	(21.84)	(10.57)	(11.36)	(10.49)	(11.34)		
AA [nmol*L^{-1}]	33.15	27.71	24.17	22.68	26.59	22.91	22.41	18.99	.415	**<.001*****
	(12.36)	11.64)	(8.25)	(8.18)	(10.36)	(7.76)	(8.53)	(5.03)		
3-HAA [nmol*L^{-1}]	20.13	18.33	24.80	37.27	16.65	24.29	27.81	39.01	.274	**<.001*****
	(12.81)	(6.41)	(9.59)	(14.67)	(7.75)	(9.60)	(14.55)	(13.40)		
3-HAA/AA ratio	.66	.75	1.11	1.81	.81	1.21	1.38	2.17	.459	**<.001*****
	(.41)	(.35)	(.57)	(.89)	(.68)	(.63)	(.79)	(.86)		
QA [nmol*L^{-1}]	456.47	412.53	429.71	430.12	373.06	369.24	359.29	354.76	.526	.301
	(98.26)	(77.34)	(70.78)	(103.01)	(81.74)	(78.67)	(76.75)	(75.40)		
QA/3-HAA ratio	29.09	27.30	26.58	12.54	26.58	19.02	17.24	10.13	.576	**<.001*****
	(15.28)	(17.50)	(29.91)	(4.02)	(11.52)	(13.71)	(9.60)	(3.97)		
Pic [nmol*L^{-1}]	70.77	55.01	70.00	71.77	47.03	49.79	52.23	57.37	.494	.277
	(38.29)	(31.91)	(59.43)	(37.77)	(15.04)	(21.91)	(19.92)	(21.11)		
Pic/3-HAA ratio	3.85	3.53	3.48	2.01	3.24	2.49	2.56	1.50	.838	**<.001*****
	(1.57)	(2.72)	(3.04)	(1.05)	(1.49)	(1.77)	(2.10)	(.37)		

Abbreviations: CON: control group; INC: intervention group; IL-6: interleukin-6; IL-10: interleukin-10; Neopt: neopterin; Trp: tryptophan; Kyn: kynurenine; KTR: kynurenine-to-tryptophan ratio (µmolL^{-1} by mmol*L^{-1}); KA: kynurenic acid; Qld: quinaldic acid; 3-HK: 3-hydroxykynurenine; XA: xanthurenic acid; AA: anthranilic acid; 3-HAA: 3-hydroxyanthranilic acid; QA: quinolinic acid; Pic: picolinic acid. T_0-T_1: all participants completed 50 min continuous walking/cycling at 55% heart rate reserve (HR_R)). Randomisation to CON/INC was performed after 10 weeks (T_1). CON participants continued 50 min continuous walking/cycling at 55%HR_R for 16 weeks (T_1-T_3). INC participants completed 50 min continuous walking/cycling at 70% HR_R for 8 weeks (T_1-T_2) and high-intensity interval training (4 x 4 min at 95% HR_R) for 8 weeks (T_2 to T_3). KTR is given in µmol*L^{-1} by mmol*L^{-1}. 3-HAA/AA ratio, QA/3-HAA ratio, and Pic/3-HAA ratio are given in nmol*L^{-}1 by nmol*L^{-1}. Values are reported as mean (SD). For IL-6, IL-10, Neopt, KTR, XA, 3-HAA/AA ratio, QA/3-HAA ratio, Pic, and Pic/3-HAA ratio, Greenhouse-Geisser corrected values are provided. Stars indicate significant time*group interactions or main effects of time at a .05 level (*), .01 level (**), or .001 level (***).*

References

1. Reuter, M. *et al.* Effects on cardiorespiratory fitness of moderate-intensity training vs. energy-matched training with increasing intensity. *Front. Sports Act. Living* **5**, 1298877 (2023).
2. Lima, S., Khristoforov, R., Momany, C. & Phillips, R. S. Crystal Structure of *Homo sapiens* Kynureninase’. *Biochemistry* **46**, 2735–2744 (2007).
3. Phillips, R. S. Structure, mechanism, and substrate specificity of kynureninase. *Biochim. Biophys. Acta BBA - Proteins Proteomics* **1814**, 1481–1488 (2011).
4. Wang, Y., Liu, K. F., Yang, Y., Davis, I. & Liu, A. Observing 3-hydroxyanthranilate-3,4-dioxygenase in action through a crystalline lens. *Proc. Natl. Acad. Sci.* **117**, 19720–19730 (2020).
